# In Vitro Three-Step Technique Assessment of a Microencapsulated Phytosynbiotic from Yanang (*Tiliacora triandra*) Leaf Extract Fermented with *P. acidilactici* V202 on Nutrient Digestibility, Cecal Fermentation, and Microbial Communities of Broilers

**DOI:** 10.3390/vetsci12100956

**Published:** 2025-10-05

**Authors:** Manatsanun Nopparatmaitree, Noraphat Hwanhlem, Atichat Thongnum, Juan J. Loor, Tossaporn Incharoen

**Affiliations:** 1 Department of Agricultural Science, Faculty of Agriculture, Natural Resources and Environment, Naresuan University, Phitsanulok 65000, Thailand; nopparatmaitree_m@silpakorn.edu (M.N.); noraphath@nu.ac.th (N.H.); 2 Department of Animal Science and Fishery, Faculty of Sciences and Agricultural Technology, Rajamangala University of Technology Lanna (Phitsanulok Campus), Phitsanulok 65000, Thailand; atichat_t@rmutl.ac.th; 3 Department of Animal Sciences, Division of Nutritional Sciences, University of Illinois, Urbana, IL 61801, USA; jloor@illinois.edu

**Keywords:** phytosyntobiotics, *Pediococcus acidilactici*, Yanang (*Tiliacora triandra*), in vitro gastrointestinal simulation, nutrient digestibility, cecal fermentation, microencapsulation, broiler nutrition

## Abstract

The poultry industry requires sustainable strategies to improve gut health and nutrient utilization while reducing antibiotic use. This study assessed the effects of dietary supplementation with a microencapsulated phytosynbiotic from Yanang (*Tiliacora triandra*) leaf extract fermented with *Pediococcus acidilactici* V202 (YEP) on broiler ileal digestibility, microbial viability, and cecal fermentation using an in vitro gastrointestinal simulation model. Six YEP inclusion levels (0–2.5%) were tested. Results revealed significant improvements in ileal dry matter and gross energy digestibility and enhanced survival and proliferation of beneficial lactic acid bacteria in the ileum. Increased gas production, lactic acid, and volatile fatty acid concentrations, including acetate, propionate, and butyrate, indicated that cecal fermentation was enhanced in a dose-dependent manner. Moderate YEP levels optimized fermentation speed and butyrate synthesis, while higher levels enhanced total gas and acetate production. YEP also shifted the cecal microbiota toward a healthier profile, enhancing Lactobacillaceae counts and the Lactobacillaceae-to-Enterobacteriaceae ratio. Overall, protective microencapsulation, synergistic phytochemical interactions, and balanced nutrient supply had positive effects at the gut level. Thus, the data highlight YEP as a promising synbiotic feed additive that can enhance nutrient utilization, microbial balance, and gut health in broilers, warranting future in vivo validation.

## Simple Summary

This study evaluated a novel microencapsulated phytosynbiotic from Yanang (*Tiliacora triandra*) leaf extract fermented with *Pediococcus acidilactici* V202 on broiler nutrient digestibility, gut fermentation, and microbial community using an adapted in vitro model. YEP supplementation improved dry matter and gross energy digestibility, supported the viability and proliferation of beneficial ileal bacteria, and enhanced cecal fermentation by increasing gas, lactic acid, and volatile fatty acid production. Microbial profiling revealed a shift toward higher Lactobacillaceae abundance and improved balance relative to Enterobacteriaceae. These findings demonstrate YEP’s potential as a sustainable synbiotic feed additive that promotes gut health and nutrient utilization in poultry, offering an effective antibiotic alternative to enhance broiler productivity and welfare.

## 1. Introduction

Global climate change, largely driven by greenhouse gas (GHG) emissions, poses a significant challenge to sustainable livestock production. Although broiler production is more efficient in terms of GHG emissions per unit of protein compared with ruminants, its vast scale contributes considerably to environmental concerns. Feed production alone accounts for up to 80% of GHG emissions in broiler systems, with additional emissions arising from manure management and cecal fermentation [1]. Beyond contributing to global warming, the major greenhouse gases (CO_2_, CH_4_, and N_2_O) create widespread impacts including nutrient losses, ecosystem disruption, and economic burdens from pollution and regulatory costs [2]. To address climate resilience, the poultry industry must improve nutrient utilization and reduce emissions. As such, reliable in vitro models to predict feed digestibility and fermentation are essential for optimizing feed formulations that support sustainable, low-emission broiler production [3].

Traditional in vivo feed evaluation methods, while accurate, are costly, time-consuming, and limited by ethical constraints. Gas production measurement techniques often require expensive equipment, limiting their routine use for broiler feed assessment [4]. In response, we have developed a novel three-step in vitro technique simulating broiler gastrointestinal digestion and fermentation, integrating enzymatic digestion, cecal fermentation, and gas production kinetics. This approach models the upper and lower gastrointestinal tract processes, allowing simultaneous assessment of nutrient digestibility and microbial fermentative capacity [5,6]. Gas kinetics analysis employs the [7] model, which has been extensively adapted in ruminant nutrition studies. This low-cost, reproducible method provides a biologically relevant alternative to in vivo trials, facilitating feed evaluation that focuses on enhancing productivity and reducing environmental impacts 8. In addition, the in vitro three step technique aligns with the principles of the 3Rs (Replacement, Reduction, and Refinement), thereby supporting ethical standards in animal experimentation [9].

Phytobiotic such as medicinal herbs and plants are well-recognized as promising antibiotic alternatives, benefiting gut microbiota and animal performance [10]. Yanang, (*Tiliacora triandra*), a Southeast Asian medicinal herb rich in antioxidants, beta-carotene, vitamins, and prebiotic oligosaccharides, improves gut health and modulates immune function [11,12,13]. Despite its traditional medicinal use, there is a paucity of research on Yanang’s effects on broiler nutrient digestibility and cecal fermentation.

*Pediococcus acidilactici* is a resilient probiotic that enhances gut health by stabilizing microbial communities, improving nutrient absorption, and supporting growth performance in broilers [14]. Its utilization results in reduced pathogen load, enhanced feed efficiency, decreased environmental nutrient excretion, and reduced feed costs, all of which represent critical attributes for sustainable, antibiotic-free poultry production systems [15]. Continuous research is needed to optimize its dosing and commercial application.

Due to harsh acidic and enzymatic conditions, maintaining probiotic viability through feed processing, storage, and gastrointestinal passage is challenging [16]. Recent advances in microencapsulation have demonstrated that natural protective matrices, particularly antioxidant-enriched Yanang leaf extract combined with prebiotic wheat bran carriers, improve probiotic stability significantly and enable controlled intestinal targeting [17,18]. Freeze-drying preserves the structural integrity and biological activity of both probiotics and phytochemicals, enhancing stability during storage and digestion [19,20].

Phytosynbiotics, a class of additives combining phytobioactive compounds with synbiotics (probiotics and prebiotics), offer synergistic benefits that improve gut health, immune response, and nutrient utilization, especially in antibiotic-free systems [21]. These additives align with rising consumer demand for natural and environmentally responsible feed solutions. The phytosynbiotic formulation developed in this study is distinguished by its use of Yanang leaf extract, which is notably rich in bioactive polyphenolic compounds exhibiting strong antioxidant and antimicrobial properties unique to this Southeast Asian plant species [12,22]. This extract was fermented with *P. acidilactic*, a probiotic strain well-documented for its resilience and beneficial effects on poultry gut health and nutrient utilization [23,24]. The formulation is further enhanced by microencapsulation within a wheat bran matrix, offering protection to the probiotic cells during feed processing and gastrointestinal transit, thereby improving their survival and functional delivery [25]. This integrated approach addresses limitations in existing phytobiotic or synbiotic products that often lack synergy between phytochemical and probiotic components or fail to ensure probiotic viability.

Regarding the in vitro model, our three-step digestion and fermentation technique simulates sequential phases of the broiler gastrointestinal tract, including enzymatic digestion with pepsin and pancreatin, followed by cecal microbial fermentation, and final assessment of gas production kinetics and microbial community shifts. This method surpasses the capabilities of conventional static or biphasic in vitro models by more accurately capturing the dynamic interactions between nutrients and microbiota under poultry-relevant physiological conditions [26,27]. Together, these novel components position our study as a significant advancement in sustainable poultry nutrition science. Thus, this research aimed to determine the effects of dietary supplementation with a microencapsulated phytosynbiotic from Yanang leaf extract fermented with *P. acidilactici* V202 (YEP) in broiler diets on in vitro nutrient digestibility and probiotic viability following gastrointestinal transit. Additionally, the study evaluated cecal fermentation dynamics encompassing gas kinetics, volatile fatty acid (VFAs) profiles, and microbial community composition using the developed gastrointestinal simulation model.

## 2. Materials and Methods

### 2.1. Animal Ethics

This study was conducted at the Animal Nutrition Laboratory, Faculty of Agriculture, Natural Resources and Environment, Naresuan University (Phitsanulok, Thailand). All experimental procedures were approved by the Naresuan University Animal Care and Use Committee (Approval ID: 68 01 008).

### 2.2. Phytosynbiotic Prototype

**Figure 1:**
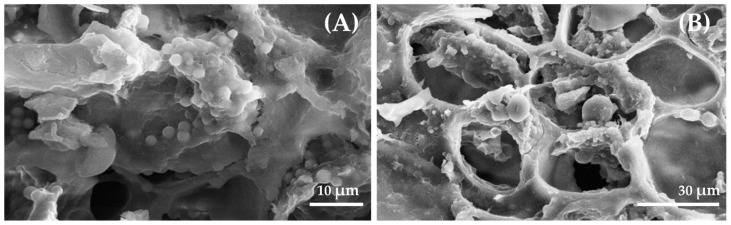
Scanning electron microscopic images of the outer surface characteristics of YEP (**A**) and the entrapment of *Pediococcus acidilactici* V202 within wheat bran porous network (**B**).

The phytosynbiotic prototype, designated as YEP, was produced by extracting and pasteurizing Yanang leaves, followed by fermentation of the extract with *P*. *acidilactici* V202 at an inoculation level of 1.5% (*v*/*v*) and incubation at 37 °C for 24 h under anaerobic conditions. The fermented extract was then homogenized with wall material, loaded into pretreated wheat bran pores under mild vacuum, frozen at −18 to −20 °C for 24 h, and lyophilized at 0.5 mbar for 48 h to yield a stable microencapsulated powder stored at 4 °C until use. The formulation achieved the viability of *P*. *acidilactici* V202 after encapsulation remained >97%, indicating excellent probiotic survival. The bulk density of the resulting material was >35 g/100 mL. Phytochemical profiling revealed a total phenolic content of >16 mg GAE/g and tannic acid at >12 mg/g, which are key contributors to the bioactivity of YEP. The morphology and surface characteristics of the YEP product was examined using scanning electron microscopy (Figure 1), which confirmed the successful encapsulation, where panel A depicts the outer surface characteristics of YEP and panel B demonstrates the entrapment of *P*. *acidilactici* V202 within the wheat bran porous network. These findings substantiate the formation of an integrated bioactive-carrier complex with enhanced protective characteristics.

### 2.3. Experimental Design

In this experiment, thirty in vitro bottles were allocated according to a completely randomized design, with six dietary treatments each with five replications. The treatments were as follows:
Treatment 1 = Control diet without supplementation (basal diet)Treatment 2 = Control diet + 0.50% YEPTreatment 3= Control diet + 1.00% YEPTreatment 4 = Control diet + 1.50% YEPTreatment 5= Control diet + 2.00% YEPTreatment 6= Control diet + 2.50% YEP

Experimental broiler diets for the grower period (22–42 days of age) were designed to meet [28] nutritional requirements and subsequently ground to achieve a uniform 2 mm particle distribution. The chemical composition of the diet was analyzed for dry matter (DM), organic matter (OM), crude protein (CP), crude fiber (CF), ether extract (EE), and gross energy (GE), following the methods outlined by the Association of Official Analytical Chemists (AOAC) [29].

### 2.4. In Vitro Ileal Nutrient Digestibility and Post-Digestion Microbial Community Responses

The in vitro ileal nutrient digestibility of experimental diets (*n* = 30) was determined using a two-step enzymatic digestion procedure adapted from [29] following the method of [30]. Approximately 0.5 g of finely ground (1 mm) feed sample was incubated with 10 mL of a pepsin solution, prepared by dissolving 0.1 g of porcine pepsin in 0.2 M HCl (pH 2.0). After the pepsin digestion phase, the pH was adjusted to 6.8 using 1 M HCl or 1 M NaOH. Subsequently, 1 mL of a freshly prepared pancreatin solution (0.5 mg pancreatin in 10 mL of 0.2 M phosphate buffer, pH 6.8) was added to simulate intestinal digestion. Proximate analysis of dietary formulations and digesta samples was conducted to determine DM, OM, CP, CF, and EE, following the methods outlined by the AOAC [29]. GE content was determined using a bomb calorimeter (LECO AC-500, LECO Corporation, St. Joseph, MI, USA), with calibration conducted using benzoic acid as a standard, according to standardized protocols [29].

True digestibility coefficients were calculated based on nutrient disappearance from the incubation residue according to [29]. The in vitro digestibility coefficients were determined by calculating the difference between the initial sample values and the undigested residue values, with adjustments made for DM content based on a blank sample included in each experimental series according to [31].

Following completion of the in vitro ileal digestibility assay, 5 mL aliquots were aseptically withdrawn from each vaccine bottle and immediately transferred into 45 mL of sterile normal saline solution (0.85% (*w*/*v*) NaCl) to obtain a 10^−1^ suspension. Serial 10-fold dilutions were prepared using sterile NSS, following the standard dilution procedure previously described by [32]. Culturable microorganisms were enumerated by plating appropriate dilutions on both non-selective and selective media. Total viable count (TVC) was enumerated by plating appropriate dilutions on Plate Count Agar (PCA) using spread-plate techniques, and plates were incubated inverted at 30–37 °C for 24 h as described by [33]. Lactobacillaceae were enumerated on de Man, Rogosa and Sharpe (MRS) agar (Merck KGaA, Darmstadt, Germany) and incubated under microaerobic or anaerobic conditions at 30–37 °C for 24 h, following [34]. Enterobacteriaceae were enumerated on MacConkey agar (bioMérieux, Marcy-l’Étoile, France) to distinguish lactose-fermenting from non-fermenting colonies, with plates incubated aerobically at 30–37 °C for 24 h, following [35]. Plates yielding 30–150 colonies were considered for quantification. Colony-forming units per milliliter (CFU/mL) were calculated based on the colony count and the corresponding dilution factor, and the survival rate (%) through the simulated gastrointestinal tract (GIT) was calculated from log_10_-transformed data as: Specific growth rate of microorganisms (h^−1^) was calculated using the following equation: Growth rate (h^−1^) = ((log_10_ N_t_ − log_10_N_0_) × 2.303)/(t − t_0_)), where Nt: number of bacteria at time point “t”, N_0_: number of bacteria at the initial time point (time point “0”), t: duration of observing time, and t_0_: initial time point (time point “0”) following the method of Nunpan et al. [36].

### 2.5. In Vitro Cecal Fermentation, Metabolite Profiles, and Microbial Community Analysis

**Figure 2:**
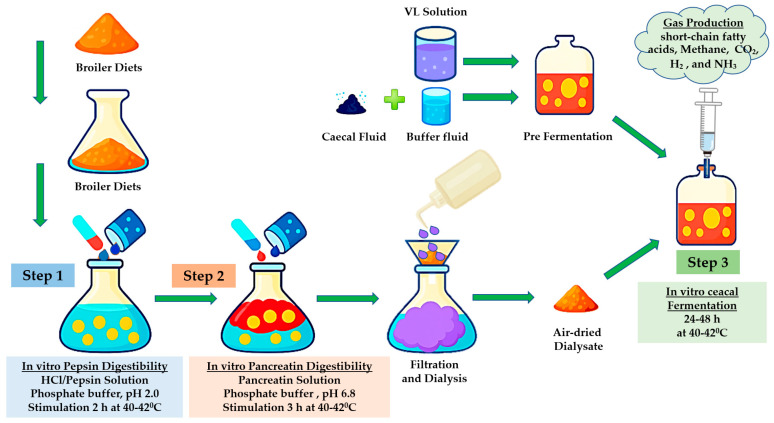
Schematic diagram of the three-step in vitro technique used to predict nutrient digestibility and cecal fermentation in broilers.

The in vitro cecal fermentation was evaluated by monitoring gas production, short-chain fatty acid (SCFAs) production, degradation kinetics, and microbial population dynamics using inoculum derived from samples of chicken cecal contents (Figure 2). Ten twenty-one-day-old male Ross 308 broilers of uniform body weight were selected from the experimental chicken farm at Naresuan University and humanely sacrificed following institutional ethical guidelines to ensure minimal distress and suffering. Prior to euthanasia, the birds were maintained on a standard corn-soy diet and monitored to ensure they were clinically healthy, with no history of antibiotic administration. This management ensured that the birds were in optimal physiological condition for cecal sample collection. Whole cecal contents were diluted 1:10 (*w*/*w*) in phosphate-buffered saline (PBS; 0.1 mol/L, pH 7.4) under anaerobic conditions prior to analysis. In vitro fermentations were carried out in 100 mL serum bottles sealed with rubber stoppers, each containing 0.3 g of digesta sample and 45 mL of sterile Viande Levure medium. The medium composition was modified from [37] and consisted of beef extract, yeast extract, glucose, tryptose, L-cysteine hydrochloride, NaCl, hemin, vitamin K, resazurin, and trace elements [30]. Prior to inoculation, media were flushed with nitrogen gas to ensure anaerobic conditions and then sterilized by autoclaving at 121 °C for 15 min. Each bottle was inoculated with 5 mL of freshly prepared slurry, diluted 1:10 (*w*/*w*) with pH-adjusted buffer (6.18–6.50), using a sterile 5 mL syringe. In vitro fermentations were carried out at 42 °C for 24 h under anaerobic conditions (Bactron 300, Sheldon Manufacturing, Cornelius, North Carolina, USA) [30]. Gas production was measured at 0, 2, 4, 6, 8, 10, 12, 14, 16, 18, 20, 22, and 24 h post-incubation using a glass syringe, following the method of [38]. The cumulative gas production data were fitted to the kinetic model of Y = a + b (1 − e^−ct^) [7]. where Y is the volume of gas produced (mL) at incubation time t (h), a represents gas production from the rapidly fermentable (upper gut digestible) fraction, b denotes gas production from the slowly fermentable (cecal fermentation) fraction, c is the rate constant of gas production for fraction b, and (a + b) indicates the potential extent of gas production [30]. Parameter estimation was performed using nonlinear regression analysis of the experimental gas production data as described by [39].

Lactic acid and VFAs were determined after 24 h of in vitro fermentation. A 1 mL aliquot of the fermentation fluid was aseptically withdrawn and centrifuged at 10,000× *g* for 10 min at 4 °C. The clear supernatant was collected and stored at −20 °C until subsequent quantification of SCFAs and lactic acid. The SCFAs (acetate, propionate, butyrate, and valeric acid) and lactic acid were analyzed using a gas chromatograph (Agilent 7890B, Agilent Technologies, Santa Clara, CA, USA) fitted with a CP-Sil 5 CB fused silica capillary column (0.32 mm × 25 m) and a flame ionization detector (FID). The injector, column oven, and detector temperatures were set according to the optimized parameters described by [40]. Nitrogen was used as the carrier gas at a constant flow rate. Internal standards consisted of 4-methylvaleric acid for SCFAs and fumaric acid for lactic acid (both from Alfa Aesar, Heysham, Lancashire, UK). Quantification was based on calibration curves generated from authentic standards, and final concentrations were calculated following a modified method by [41].

After the in vitro digestibility assay was completed, 5 mL portions were aseptically collected from each container and promptly mixed with 45 mL of sterile NSS to yield a 10^−1^ suspension. Subsequently, 10-fold serial dilutions were prepared in sterile NSS following the standard method outlined by [32]. Enumeration of viable microorganisms was carried out by spreading or pouring suitable dilutions onto both selective and non-selective agar media. For TVC, aliquots were plated on Plate Count Agar (PCA) and incubated at 30–32 °C for 24 h, according to the procedure of [33]. Lactobacillaceae populations were assessed using de Man, Rogosa and Sharpe (MRS) agar, incubated under microaerophilic or anaerobic conditions at 30–37 °C for 24 h, as described by [34]. Enterobacteriaceae were enumerated on MacConkey agar to distinguish lactose-fermenting from non-fermenting colonies, with plates incubated aerobically at 30–37 °C for 24 h, following [35]. Only plates containing 30–150 colonies were used for quantification. The number of colony-forming units per milliliter (CFU/mL) was calculated from the colony counts and the corresponding dilution factors, and the ratio of Lactobacillaceae to Enterobacteriaceae populations was determined accordingly.

### 2.6. Statistical Analysis

Data were analyzed using analysis of variance (ANOVA) in a completely randomized design (CRD) according to the model: y_ij_ = μ + T_i_ + ε_ij_, where y_ij_ represents the observation for the ith treatment (i = 1–6) and jth replicate (j = 1–5), μ is the overall mean, T_i_ is the fixed effect of YEP supplementation at 0%, 0.50%, 1.00%, 1.50%, 2.00%, and 2.50%, and ε_ij_ is the random error term. Differences were considered statistically significant at *p* < 0.05. When significant effects were detected, treatment means were separated using Tukey’s Honestly Significant Difference (HSD) test. Orthogonal contrasts were performed to compare specific treatment groups, including Control vs. low level of dietary 0.50 and 1.00% YEP supplementation (L-YEP) and Control vs. high level of dietary 1.50%, 2.00%, and 2.50% YEP supplementation (H-YEP). Furthermore, orthogonal polynomials were applied to test linear, quadratic, and cubic trends across increasing dietary levels of YEP. Comparisons between control and individual levels were further evaluated using predetermined contrasts. This approach is suited to assessing dose–response relationships. All statistical analyses were performed using orthogonal polynomials in R (version 4.3.3) with the package ‘agricolae’ [42].

## 3. Results

### 3.1. Nutritional Analysis of Experimental Diets

The nutrient composition of broiler diets supplemented with increasing levels of YEP during the grower period had minimal variation across treatments (Table 1). All diets contained comparable DM (91.31–92.13%) and CP (21.36–21.91%) contents. The EE and CF levels were similar among groups, as was GE (4012.30–4089.60 kcal/kg). This indicated that YEP supplementation up to 2.50% did not markedly alter the basic nutrient profile of broiler diets.

### 3.2. Assessment of YEP Supplementation on In Vitro Ileal Digestibility

The impact of dietary YEP supplementation on in vitro ileal digestibility is presented in Table 2. YEP supplementation enhanced (*p* = 0.001) DM digestibility, with the dietary YEP supplementation inclusion levels demonstrating superior responses relative to the control group. While CP digestibility remained unaffected (*p* = 0.184), numerical increases were evident across all YEP treatments. There was a trend (*p* = 0.088) toward improvement of EE digestibility, most notably at the 1.00% and 1.50% inclusion rates. The CF digestibility did not differ (*p* = 0.473), whereas, compared with the unsupplemented control, GE digestibility was greater (*p* = 0.033) across all YEP supplementation levels.

Orthogonal polynomial contrasts demonstrated that both moderate (0.50–1.00%) and elevated (1.50–2.50%) YEP inclusion rates improved (*p* < 0.05) DM, EE, and GE digestibility relative to the control treatment. Dose–response relationships were characterized by significant linear and quadratic effects for DM digestibility (*p* = 0.006 and *p* = 0.002, respectively) and GE digestibility (*p* = 0.008 and *p* = 0.058, respectively). Additionally, there was a quadratic effect (*p* = 0.036) on EE digestibility, indicating optimal inclusion levels within the tested range.

### 3.3. Supplementation of YEP Affected Microbial Community Responses After In Vitro Digestion

In vitro assessment of dietary YEP supplementation on TVC and Lactobacillaceae bacterial populations is detailed in Table 3. TVC enumeration was significantly enhanced (*p* = 0.003) by YEP inclusion, with peak densities achieved at 1.50% and 2.50% supplementation levels (8.826 and 8.852 log CFU/mL) compared with the unsupplemented control (8.466 log CFU/mL). Lactobacillaceae populations had a pronounced response to YEP supplementation (*p* < 0.001), exhibiting maximum counts at 1.50% and 2.50% inclusion rates (8.654 and 8.296 log CFU/mL, respectively) relative to the basal treatment (8.414 log CFU/mL). Bacterial growth rate parameters revealed optimal TVC survival in the 0.50% YEP treatment (0.36 h^−1^), while Lactobacillaceae viability was maximized at 2.50% YEP inclusion (0.58 h^−1^). Compared with the control, orthogonal polynomial analysis confirmed that both moderate (0.50–1.00%) and elevated (1.50–2.50%) YEP supplementation increased microbial populations, with significant linear dose–response relationships observed for both bacterial groups.

### 3.4. Effect of Dietary YEP on In Vitro Cecal Fermentation

**Figure 3:**
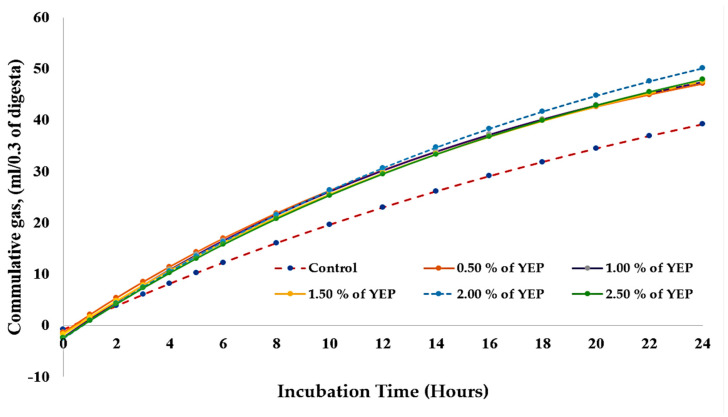
Alterations in gas production over incubation time during in vitro cecal fermentation by broiler microbiota following dietary YEP supplementation.

The impact of dietary YEP supplementation on cumulative gas production during in vitro cecal fermentation across various incubation intervals is presented in Table 4 and Figure 3. Relative to the control group, YEP supplementation enhanced gas production throughout all evaluated incubation periods (4–24 h) (*p* < 0.001). During the initial 4 h incubation period, cumulative gas production exhibited a range from 8.13 mL in the control to 11.41 mL in the 0.50% dietary YEP group. Following 24 h of incubation, maximal gas production was recorded with the 2.00% dietary YEP treatment (50.11 mL), followed by the 1.50% YEP group (49.80 mL), whereas the control group yielded 39.22 mL.

Compared with the control, orthogonal contrast analysis revealed that both the lower (0.50–1.00% dietary YEP groups) and higher (1.50–2.50% dietary YEP groups) levels of YEP supplementation enhanced (*p* < 0.001) gas production across all incubation intervals. Comparative analysis between the lower and higher YEP supplementation levels revealed statistically significant differences at most time points, with the exception of the 4 h incubation period (*p* = 0.059). Orthogonal polynomial regression analysis revealed significant linear and quadratic response patterns across all incubation periods (*p* < 0.001), indicating a concentration-dependent relationship in gas production kinetics.

### 3.5. Dietary YEP Affects Degradation Kinetics During In Vitro Cecal Fermentation by Broiler Microbiota

The influence of dietary YEP on degradation kinetics during in vitro cecal fermentation by broiler microbiota is presented in Table 5. The YEP supplementation exerted significant effects on all kinetic parameters (P, a, b, c, d (*p* < 0.05). Maximum cumulative gas production (P) was achieved with the 2.00% YEP groups (45.38 mL), followed by the 1.50% dietary YEP group (44.84 mL), both substantially exceeding the control group (37.70 mL). Gas production attributed to the upper gut digestible fraction (a) exhibited a declining trend with increasing dietary YEP inclusion, ranging from −0.85 mL in the control to −2.57 mL in the 2.00% dietary YEP group. Conversely, gas production derived from the cecal fermentation fraction (b) demonstrated a positive response to YEP supplementation, with the highest values recorded with the 2.00% dietary YEP group (80.33 mL). The rate constant governing cecal fermentation kinetics (c) reached optimal levels with the 0.50% and 1.00% dietary YEP groups (0.053%/h), indicating accelerated fermentation dynamics at moderate supplementation concentrations. The theoretical maximum gas production potential (d = a + b) was maximized with the 2.00% YEP treatment (82.91 mL).

Orthogonal contrast analysis revealed that, relative to the control, both moderate (0.50–1.00% YEP) and elevated (1.50–2.50% YEP) supplementation levels enhanced (*p* < 0.05) cumulative gas production and associated kinetic parameters. Distinct differentiation was observed between moderate and elevated YEP levels for multiple parameters (P, a, b, d). Orthogonal polynomial regression analysis demonstrated linear and quadratic response relationships for P, a, and c parameters, substantiating concentration-dependent modifications in gas production kinetics. Cubic polynomial effects achieved statistical significance for b, c, and d parameters, suggesting subtle nonlinear response patterns at discrete supplementation concentrations.

### 3.6. Supplementation of YEP Stimulates Lactic Acid and VFA Production During In Vitro Cecal Fermentation

The effect of dietary YEP supplementation on lactic acid and VFA concentrations during in vitro cecal fermentation is shown in Table 6. Lactic acid concentrations exhibited increases following YEP supplementation (*p* < 0.001), ranging from 12.77 mM/L in the control treatment to 14.73 mM/L in the 2.50% YEP treatment group. Total VFA accumulation had a corresponding increase, with maximum concentrations recorded with the 2.50% dietary YEP group (28.33 mM/L) relative to the control (22.14 mM/L). Among individual VFA, acetate (C2) and propionate (C3) concentrations were enhanced by YEP supplementation, achieving maximum concentrations of 21.71 mM/L (C3) with 2.00% dietary YEP inclusion and 28.33 mM/L (C2) with 2.50% dietary YEP group (*p* < 0.001). Butyrate (C4) concentrations reached optimal levels in the 1.50% dietary YEP group (4.98 mM/L), while valerate (C5) and lactic acid had a comparable increasing response pattern as the level of supplementation increased.

Orthogonal contrast analysis demonstrated that, compared with the control, both moderate (0.50–1.00% dietary YEP) and elevated (1.50–2.50% dietary YEP) supplementation levels significantly enhanced lactic acid, total VFAs, acetate, and propionate concentrations (*p* < 0.01). Additional statistically significant differences were observed between moderate and elevated supplementation levels for the majority of measured parameters. Orthogonal polynomial regression analysis revealed significant linear response relationships for lactic acid, total VFA, C2, C3, and C4 concentrations (*p* < 0.01), confirming concentration-dependent increases in VFA biosynthesis.

### 3.7. Alterations of Microbial Community Dynamics During In Vitro Cecal Fermentation by Broiler Microbiota

The impact of dietary YEP supplementation on microbial community dynamics during in vitro cecal fermentation by broiler microbiota is presented in Table 7. Total bacterial counts were enhanced (*p* = 0.001) following dietary YEP supplementation, with cell densities ranging from 9.03 log CFU/mL in the control to 9.22 log CFU/mL in the 1.00% dietary YEP group. Lactobacillaceae populations exhibited significant proliferation (*p* < 0.001) in response to dietary YEP inclusion, with peak bacterial densities recorded with the 0.50% dietary YEP group (8.64 log CFU/mL). Enterobacteriaceae populations were unaffected by dietary treatment (*p* = 0.106), whereas the Lactobacillaceae-to-Enterobacteriaceae ratio (L:E) was enhanced (*p* < 0.001), indicating a microbiome shift toward beneficial bacterial populations within the cecal ecosystem.

Orthogonal contrast analysis revealed that relative to the control treatment both moderate (0.50–1.00% YEP) and elevated (1.50–2.50% YEP) supplementation levels enhanced (*p* < 0.01) total bacterial counts, Lactobacillaceae populations, and L:E ratios. There were no statistically significant effects on total bacterial counts or L:E ratios between moderate and elevated YEP supplementation levels. Orthogonal polynomial regression analysis indicated significant linear response relationships for Lactobacillaceae populations and L:E ratios (*p* < 0.01), along with quadratic and cubic polynomial effects for total bacterial counts and Lactobacillaceae populations, suggesting concentration-dependent microbial proliferation with subtle nonlinear response patterns.

## 4. Discussion

Dietary supplementation with YEP enhanced the viability of both total and Lactobacillaceae or lactic acid bacteria (LAB) following in vitro ileal digestion. The most pronounced beneficial effects were observed at moderate inclusion levels (0.50–1.50% YEP), highlighting an optimal range for sustaining microbial growth rate. This effect is primarily attributed to the protective role of microencapsulation, which shields *P. acidilactici* V202 from adverse conditions in the upper gastrointestinal tract, including low pH, digestive enzymes, and osmotic stress. As a result, controlled probiotic release and survival in the ileum are facilitated, where the environment is more favorable for probiotic activity [43,44]. Additionally, the enhanced survival of LAB may be attributed to synergistic interactions between the encapsulated probiotic and the bioactive phytochemicals present in Yanang, particularly its mucilaginous components [12]. These results align with [45], who demonstrated that natural carriers and encapsulating materials, including xanthan gum (2%), maltodextrin (1%), alginate (10:1), cocoa powder combined with sodium alginate and fructooligosaccharides (10:1:2), alginate with Persian gum (1.5%:0.5%), and inulin (2%), effectively enhance probiotic delivery and viability. Such synergistic effects are thought to create a favorable microenvironment that preserves probiotic integrity and functionality during both storage and gastrointestinal transit. In the present study, wheat bran was employed as the encapsulating matrix due to its natural prebiotic properties and high porosity, which facilitate efficient probiotic load and release. Similarly [46] has shown that wheat bran are effective carrier materials capable of protecting *Lactobacillus casei* and enhancing the viability of the probiotic cells.

Furthermore, microencapsulation technology is widely recognized for its ability to maintain probiotic viability under adverse stressors, including heat, humidity, and the harsh physicochemical conditions of the gastrointestinal tract [47]. Encapsulation provides a protective barrier around probiotics, consisting of either plant-derived cell wall components such as wheat bran [48] or specialized polymers. This barrier shields the bacteria from physical and chemical stressors that would otherwise compromise cell viability. Empirical evidence demonstrates that encapsulated probiotics consistently exhibit greater survival under simulated gastric and intestinal conditions than their non-encapsulated counterparts. For example, *Lactobacillus rhamnosus* encapsulated within alginate-xanthan microcapsules had improved resistance to digestion [49]. Similarly, microencapsulation of *Lacticaseibacillus paracasei* with extracts from *Ficus pumila* seeds enhanced bacterial survival and stability [34]. Similarly, encapsulation provides a protective barrier around probiotics, consisting of either plant-derived cell wall components such as wheat bran [48] or specialized polymers. Recent findings have confirmed that wheat bran serves as an effective carrier matrix for immobilizing *L. casei* ATCC 393 cells, enabling the production of durable freeze-dried immobilized biocatalysts suitable for industrial applications [50]. Moreover, employing wheat bran for cell immobilization with *L. casei* and *L. bulgaricus* has been shown to significantly enhance probiotic survivability when exposed to simulated gastric juice at pH 3.0, further supporting the benefits of cereal-derived fibers as protective delivery systems [51].

Natural encapsulating materials and cereal-derived matrices, such as wheat bran, can enhance probiotic survival by providing fermentable carbon sources and modulating the surrounding pH. These conditions not only support the stability of encapsulated microbes but also influence the release dynamics within the gastrointestinal tract. Bioactive phytochemicals in Yanang further contribute by exerting selective antimicrobial activity [52] while promoting LAB growth, creating an environment favorable for probiotic proliferation. Polyphenolic compounds from Yanang leaf extract may additionally suppress non-beneficial bacteria [53] and provide metabolic substrates that enhance the growth of beneficial microbes. A quadratic response was observed with higher inclusion levels, where excessive supplementation (2.50% YEP) slightly reduced viability, potentially due to antimicrobial overload or nutrient saturation inhibiting bacterial balance. Overall, microencapsulation of *P. acidilactici* V202 with Yanang leaf extract in wheat bran effectively protects probiotics and promotes their delivery to the ileum, where they can exert metabolic and immunological benefits. This approach highlights the potential of YEP as a synbiotic feed additive to support gut microbial balance and improve nutrient utilization in poultry, consistent with current advancements in synbiotic applications in animal nutrition.

The improvement in nutrient digestibility observed with YEP supplementation may be explained by the synergistic actions of *P. acidilactici* and the bioactive compounds present in Yanang leaf extract. Probiotics and synbiotics are known to enhance enzymatic activity, reduce anti-nutritional factors, and modulate intestinal microbiota, collectively leading to improved nutrient utilization [54]. In parallel, phytochemicals derived from Yanang, such as phenolics, flavonoids, terpenoids, fatty acids, amino acids, peptides, carbohydrates, and vitamins [55] are likely to provide prebiotic-like functions by supporting the growth of beneficial bacteria [56], thereby promoting nutrient breakdown and absorption.

Microencapsulation also plays a crucial role. By protecting probiotic viability under harsh gastrointestinal conditions and ensuring gradual release, this technique likely contributes to consistent improvements in DM and GE digestibility across different supplementation levels. Although CP and EE digestibility tended to increase, the differences were not statistically significant, which may reflect already efficient utilization of protein and fat in the substrate. Similarly, CF digestibility had a numerical but non-significant improvement, consistent with the limited fiber-degrading capacity of poultry and the lack of cellulolytic ability in *P. acidilactici* [57]. From a practical standpoint, both low and high levels of dietary YEP outperformed the control, yet no statistical differences were detected between inclusion rates. This suggests that relatively low supplementation is sufficient to improve nutrient digestibility, offering an optimal balance between cost and efficacy for feed formulation in poultry production.

The present study demonstrated that dietary supplementation with YEP influenced in vitro cecal fermentation by broiler microbiota. Feeding YEP enhanced gas production across all incubation times compared with the control, indicating robust fermentative activity. The effect was most pronounced during the early fermentation phase (4–8 h) at moderate inclusion levels (0.50–1.00%), suggesting that these doses accelerate the onset of microbial fermentation. This observation is consistent with previous reports highlighting synergistic effects between probiotics and plant-derived bioactives in enhancing fiber degradation and microbial metabolism [58]. During the latter incubation period (16–24 h), enhanced gas production likely reflected sustained utilization of both soluble and structural carbohydrates, which may be facilitated by the prebiotic properties of arabinoxylans present in wheat bran [59]. Yanang leaf extract may have further contributed by exerting selective antimicrobial effects against non-beneficial bacteria, thereby supporting the dominance of fibrolytic species [60] and lactic acid bacteria [61]. In parallel, *P. acidilactici* may have promoted enzymatic activity and microbial cross-feeding, improving carbohydrate utilization and SCFAs production [62]. Kinetic modeling confirmed that dietary YEP increased both the potential gas production (P) and the gas yield from the fermentable fraction (b), particularly at higher inclusion levels (1.50–2.00%). By contrast, the rate constant (c) was greatest at lower dosages (0.50–1.00%), indicating that moderate supplementation improves fermentation speed, whereas higher levels enhance substrate degradation. These nonlinear, dose-dependent effects are aligned with previous synbiotic studies and may reflect microbial competition or substrate saturation [63].

The VFA profiles and lactic acid production were also significantly affected by YEP supplementation. Lactic acid concentrations increased progressively with higher inclusion, peaking at 2.50%, reflecting active metabolism of LAB and efficient fermentation of wheat bran substrates. Total VFA production followed a similar trend, with acetate being the predominant metabolite. Both acetate and propionate concentrations were highest at 2.00–2.50% YEP inclusion, providing energy for the host and potentially supporting metabolic health. Butyrate exhibited a quadratic response, peaking at 1.50% dietary YEP. These results suggest that moderate supplementation may stimulate the growth of butyrate-producing fibrolytic bacteria and bifidogenic populations, whereas higher doses could suppress certain microbial groups due to excess phenolic compounds [64]. Taken together, these findings suggest that moderate YEP inclusion optimizes the gut environment for butyrate producers, whereas higher doses shift the fermentation pro-file toward acetate and propionate accumulation. This mechanistic insight supports the selection of inclusion rates that maximize desired metabolic outcomes for broiler gut health. Valerate production was only slightly affected, with moderate inclusion supporting its formation, while higher doses reducing it, likely reflecting a shift toward saccharolytic fermentation [65]. The supplied substrates, including fibers and oligosaccharides, promote the proliferation of both butyrate-producing fibrolytic bacteria and bifidogenic microbes, helping to maintain balanced microbial fermentation [60]. and leading to the production of SCFAs such as butyrate and valerate. Orthogonal contrasts and polynomial analyses confirmed both linear and nonlinear dose–response patterns, highlighting the importance of optimal inclusion levels. Low to moderate supplementation (1.00–1.50%) maximized butyrate production and fermentation rate, whereas higher inclusion (2.00–2.50%) mainly increased acetate and total VFA yields. Collectively, these findings suggest that YEP can effectively modulate cecal fermentation, enhancing lactic acid and beneficial VFA production. Thus, balancing inclusion levels is critical: moderate doses optimize butyrate formation, supporting gut integrity, while higher doses favor acetate and propionate accumulation. These in vitro results warrant in vivo validation to determine effects on growth performance, nutrient digestibility, and overall gut health in broilers.

Maintaining an optimal carbon-to-nitrogen (C/N) ratio is fundamental for efficient microbial fermentation, as it ensures adequate fermentable carbon for energy provision and sufficient nitrogen for microbial protein synthesis [66]. In the present study, wheat bran supplied fermentable fiber, whereas Yanang extract and probiotic biomass contributed nitrogenous compounds, generating a balanced substrate that favored the proliferation of fibrolytic and lactic acid bacteria [67]. Such a nutrient-balanced environment is likely to support SCFAs-producing communities over proteolytic or pathogenic bacteria, resulting in improved fermentation efficiency. Consistent with this, YEP supplementation enhanced fermentation kinetics, and the achieved C/N ratio likely optimized both the rate and extent of carbohydrate degradation by the cecal microbiota.

In addition to fermentation dynamics, YEP supplementation increased TVC and enriched Lactobacillaceae populations, leading to a higher Lactobacillaceae-to-Enterobacteriaceae (L:E) ratio. This microbial shift reflects the synergistic contributions of *P. acidilactici*, bioactive polyphenols, and dietary fiber, which collectively promote LAB growth while suppressing opportunistic Enterobacteriaceae [61]. Interestingly, quadratic response patterns were observed, with moderate YEP inclusion (0.5–1.0%) providing the most favorable effects, likely due to maintaining a balanced C/N ratio while avoiding inhibitory phytochemical levels. Further, microencapsulation technology likely protected probiotic viability and ensured their release in the cecum. Collectively, these findings position YEP as a promising synbiotic feed additive and a sustainable alternative to antibiotic growth promoters, with potential benefits for nutrient utilization, microbial modulation, and gut health in poultry.

## 5. Conclusions

Supplementation of broiler diets with phytosynbiotics through microencapsulation of *P. acidilactici* V202 combined with Yanang leaf extract in wheat bran improved in vitro ileal digestibility, particularly for DM and GE, while enhancing the proliferation and viability of beneficial ileal bacteria. Dietary YEP also stimulated cecal fermentation by increasing gas production, lactic acid concentrations, and the generation of VFAs, including acetate, propionate, and butyrate, in a dose-dependent manner. Feeding YEP altered the cecal microbial community toward a more favorable structure, reflected by higher TVC and enrichment of Lactobacillaceae, whereas Enterobacteriaceae levels were not significantly reduced. Collectively, these findings indicate that YEP supplementation can support nutrient utilization, optimize microbial fermentation, and promote a healthier intestinal microbiota in broilers. This approach highlights YEP as a promising synbiotic feed additive and a sustainable alternative to conventional antibiotic growth promoters, with potential benefits for poultry gut health and productive performance.

## Figures and Tables

**Table 1:** Nutrient composition of grower broiler diets supplemented with varying levels of YEP.

Dietary YEP Supplementation, %	Nutrient Composition ^1^				
	DM, %	CP, %	EE, %	CF, %	GE, kcal/kg
0 (Control)	91.35	21.56	5.39	4.26	4079
0.50	91.31	21.91	5.23	4.19	4033
1.00	92.02	21.71	5.60	4.51	4012
1.50	91.67	21.57	5.00	4.49	4073
2.00	92.13	21.40	5.51	3.54	4089
2.50	91.76	21.36	5.30	3.79	4069

^1^ Each nutrient composition was determined: DM = dry matter; CP = crude protein; EE = ether extract; CF = crude fiber; GE = gross energy.

**Table 2:** Assessment of YEP supplementation in a grower broiler diet on in vitro ileal digestibility.

Dietary YEP Supplementation, %	In Vitro Ileal Nutrient Digestibility ^1^, %				
	DM	CP	EE	CF	GE
0 (Control)	77.61 ^b^	80.93	81.83	42.10	82.32 ^b^
0.50	79.42 ^a^	82.10	82.77	43.93	83.29 ^a^
1.00	79.88 ^a^	81.97	83.43	42.58	83.23 ^a^
1.50	79.18 ^a^	81.77	83.10	42.53	83.30 ^a^
2.00	79.57 ^a^	82.10	82.53	42.75	83.75 ^a^
2.50	79.34 ^a^	81.83	82.87	43.63	83.35 ^a^
SEM	0.08	0.15	0.17	0.25	0.14
*p*-value	0.001	0.184	0.088	0.473	0.033
Orthogonal contrasts					
Control vs. L-YEP	0.001	0.017	0.011	0.157	0.010
Control vs. H-YEP	0.002	0.019	0.008	0.229	0.006
L-YEP vs. H-YEP	0.125	0.460	0.104	0.261	0.175
Orthogonal polynomial					
Linear	0.006	0.139	0.207	0.867	0.008
Quadratic	0.002	0.099	0.036	0.556	0.058
Cubic	0.087	0.246	0.112	0.20	0.051

^1^ In vitro ileal nutrient digestibility was assessed as: DM = dry matter; CP = crude protein; EE = ether extract; CF = crude fiber; GE = gross energy. SEM = standard error of the mean. YEP = a microencapsulation of Yanang leaf extract fermented with *Pediococcus acidilactici* V202, followed by entrapment within the porous structure of wheat bran. L-YEP = low level of dietary supplementation (0.50 and 1.00% YEP groups). H-YEP = high level of dietary supplementation (1.50%, 2.00%, and 2.50% YEP groups). ^a,b^ Different superscripts in the same column indicate significant differences (*p* < 0.05).

**Table 3:** In vitro assessment of dietary YEP supplementation on total viable count (TVC) and Lactobacillaceae populations.

	Microbial Responses (log CFU/mL)					
	TVC			Lactobacillaceae		
Dietary YEP Supplementation, %	Initial	Final	Growth Rate (h^−1^)	Initial	Final	Growth Rate (h^−1^)
0 (Control)	7.67	8.47 ^c^	0.28 ^BC^	7.38	8.41 ^b^	0.42 ^C^
0.50	7.72	8.62 ^bc^	0.36 ^A^	7.34	8.67 ^a^	0.49 ^B^
1.00	7.81	8.69 ^ab^	0.22 ^CD^	7.31	8.39 ^b^	0.52 ^AB^
1.50	7.83	8.83 ^a^	0.31 ^AB^	7.36	8.65 ^a^	0.56 ^A^
2.00	7.76	8.75 ^ab^	0.28 ^BC^	7.48	8.60 ^a^	0.48 ^B^
2.50	7.81	8.85 ^a^	0.18 ^D^	7.33	8.30 ^b^	0.58 ^A^
SEM	0.022	0.037	0.007	0.019	0.037	0.009
*p*-value	0.229	0.003	<0.001	0.090	<0.001	0.002
Orthogonal contrasts						
Control vs. L-YEP	0.152	0.016	0.285	0.255	0.032	0.001
Control vs. H-YEP	0.067	0.002	0.349	0.281	0.005	0.001
L-YEP vs. H-YEP	0.055	0.004	0.115	0.556	0.348	0.039
Orthogonal polynomial						
Linear	0.064	<0.001	0.002	0.629	0.128	0.006
Quadratic	0.187	0.124	0.027	0.776	<0.001	0.105
Cubic	0.635	0.540	0.001	0.021	0.062	0.09

SEM = standard error of the mean. YEP = a microencapsulation of Yanang leaf extract fermented with *Pediococcus acidilactici* V202, followed by entrapment within the porous structure of wheat bran. L-YEP = low level of dietary supplementation (0.50 and 1.00% YEP groups). H-YEP = high level of dietary supplementation (1.50%, 2.00%, and 2.50% YEP groups). ^A,B,C,D^ Different superscripts in the same column indicate significant difference (*p* < 0.01). ^a,b,c^ Different superscripts in the same column indicate significant differences (*p* < 0.05).

**Table 4:** Effect of dietary YEP on gas production at different incubation times during in vitro cecal fermentation by broiler microbiota.

Dietary YEP Supplementation, %	Gas Production in Different Incubation Times (mL)					
	4 h	8 h	12 h	16 h	20 h	24 h
0 (Control)	8.13 ^c^	16.04 ^b^	22.99 ^b^	29.10 ^b^	34.48 ^b^	39.22 ^d^
0.50	11.41 ^a^	21.81 ^a^	30.24 ^a^	37.06 ^a^	42.58 ^a^	47.05 ^c^
1.00	10.87 ^ab^	21.54 ^a^	30.18 ^a^	37.18 ^a^	42.85 ^a^	47.44 ^bc^
1.50	11.05 ^ab^	21.90 ^a^	30.92 ^a^	38.41 ^a^	44.63 ^a^	49.80 ^ab^
2.00	10.53 ^b^	21.49 ^a^	30.66 ^a^	38.33 ^a^	44.74 ^a^	50.11 ^a^
2.50	10.22 ^b^	20.74 ^a^	29.50 ^a^	36.77 ^a^	42.82 ^a^	47.85 ^abc^
SEM	0.245	0.463	0.614	0.714	0.775	0.811
*p*-value	<0.001	<0.001	<0.001	<0.001	<0.001	<0.001
Orthogonal contrasts						
Control vs. L-YEP	<0.001	<0.001	<0.001	<0.001	<0.001	<0.001
Control vs. H-YEP	<0.001	<0.001	<0.001	<0.001	<0.001	<0.001
L-YEP vs. H-YEP	0.059	<0.001	<0.001	<0.001	<0.001	<0.001
Orthogonal polynomial						
Linear	0.001	<0.001	<0.001	<0.001	<0.001	<0.001
Quadratic	<0.001	<0.001	<0.001	<0.001	<0.001	<0.001
Cubic	<0.001	<0.001	0.001	0.007	0.049	0.244

SEM = standard error of the mean. YEP = a microencapsulation of Yanang leaf extract fermented with *Pediococcus acidilactici* V202, followed by entrapment within the porous structure of wheat bran. L-YEP = low level of dietary supplementation (0.50 and 1.00% YEP groups). H-YEP = high level of dietary supplementation (1.50%, 2.00%, and 2.50% YEP groups). ^a,b,c,d^ Different superscripts in the same column indicate significant difference (*p* < 0.05).

**Table 5:** Impact of dietary YEP on degradation kinetics during in vitro cecal fermentation by broiler microbiota.

Dietary YEP Supplementation, %	Degradation Kinetic ^1^				
	P (mL)	a (mL)	b (mL)	c (%hour)	d (mL)
0 (Control)	37.70 ^c^	−0.85 ^a^	75.06 ^ab^	0.03 ^c^	75.91 ^abc^
0.50	41.63 ^b^	−1.44 ^a^	67.62 ^c^	0.05 ^a^	69.05 ^c^
1.00	41.87 ^b^	−2.32 ^cd^	69.37 ^bc^	0.05 ^a^	71.69 ^bc^
1.50	44.84 ^a^	−2.02 ^c^	77.17 ^a^	0.05 ^ab^	79.19 ^a^
2.00	45.38 ^a^	−2.57 ^d^	80.33 ^a^	0.04 ^b^	82.91 ^a^
2.50	43.10 ^ab^	−2.45 ^cd^	75.27 ^ab^	0.05 ^ab^	77.72 ^ab^
SEM	0.583	0.141	1.177	0.002	1.239
*p*-value	<0.001	<0.001	0.004	<0.001	0.004
Orthogonal contrasts					
Control vs. L-YEP	<0.001	<0.001	0.017	<0.001	0.048
Control vs. H-YEP	<0.001	<0.001	0.148	<0.001	0.320
L-YEP vs. H-YEP	<0.001	<0.001	0.014	0.317	0.006
Orthogonal polynomial					
Linear	<0.001	<0.001	0.014	0.004	0.005
Quadratic	<0.001	0.006	0.396	<0.001	0.557
Cubic	0.272	0.603	<0.001	<0.001	0.001

^1^ Degradation kinetic were measured as: P = gas produced at time ‘t’; a = gas production from upper gut digestible fraction; b = gas production from cecal fermentation fraction; c = gas production rate constant for cecal fermentation fraction (b); d = (a + b) potential extent of gas production. SEM = standard error of the mean. YEP = a microencapsulation of Yanang leaf extract fermented with *Pediococcus acidilactici* V202, followed by entrapment within the porous structure of wheat bran. L-YEP = low level of dietary supplementation (0.50 and 1.00% YEP groups). H-YEP = high level of dietary supplementation (1.50%, 2.00%, and 2.50% YEP groups). ^a,b,c,d^ Different superscripts in the same column indicate significant difference (*p* < 0.05).

**Table 6:** Influence of dietary YEP on lactic acid and VFAs content during in vitro cecal fermentation by broiler microbiota.

Dietary YEP Supplementation, %	Lactic Acid (mM/L)	VFAs Content (mM/L)				
		Total VFA	Acetic Acid	Propionic Acid	Butyric Acid	Valeric Acid
0 (Control)	12.77 ^e^	22.14 ^e^	16.83 ^c^	4.11 ^d^	0.73 ^c^	0.47 ^cd^
0.50	12.95 ^e^	22.70 ^e^	17.14 ^c^	4.31 ^c^	0.76 ^bc^	0.49 ^bc^
1.00	13.93 ^d^	25.80 ^d^	18.54 ^b^	4.63 ^b^	0.79 ^abc^	0.53 ^a^
1.50	14.15 ^c^	26.51 ^c^	18.87 ^b^	4.98 ^a^	0.83 ^a^	0.50 ^ab^
2.00	14.53 ^b^	27.70 ^b^	21.71 ^a^	4.69 ^b^	0.80 ^ab^	0.51 ^ab^
2.50	14.73 ^a^	28.33 ^a^	21.56 ^a^	4.32 ^c^	0.77 ^bc^	0.45 ^d^
SEM	0.167	0.525	0.455	0.082	0.009	0.011
*p*-value	<0.001	<0.001	<0.001	<0.001	0.018	0.003
Orthogonal contrasts						
Control vs. L-YEP	<0.001	<0.001	0.003	0.003	0.084	0.005
Control vs. H-YEP	<0.001	<0.001	<0.001	<0.001	0.011	0.006
L-YEP vs. H-YEP	<0.001	<0.001	<0.001	<0.001	0.003	0.015
Orthogonal polynomial						
Linear	<0.001	<0.001	<0.001	<0.001	0.020	0.484
Quadratic	0.001	0.001	0.116	<0.001	0.006	<0.001
Cubic	0.021	0.021	0.007	0.003	0.179	0.771

VFAs = volatile fatty acids. SEM = standard error of the mean. YEP = a microencapsulation of Yanang leaf extract fermented with *Pediococcus acidilactici* V202, followed by entrapment within the porous structure of wheat bran. L-YEP = low level of dietary supplementation (0.50 and 1.00% YEP groups). H-YEP = high level of dietary supplementation (1.50%, 2.00%, and 2.50% YEP groups). ^a,b,c,d,e^ Different superscripts in the same column indicate significant difference (*p* < 0.05).

**Table 7:** The effect of dietary YEP supplementation on microbial community dynamics during in vitro cecal fermentation by broiler microbiota.

Dietary YEP Supplementation, %	Microbial Community Dynamics (log CFU/mL)			
	Total Bacteria	Lactobacillaceae (L)	Enterobacteriaceae (E)	L:E Ratio
0 (Control)	9.03 ^b^	8.09 ^c^	6.90	1.17 ^c^
0.50	9.21 ^a^	8.64 ^a^	6.84	1.26 ^a^
1.00	9.22 ^a^	8.60 ^ab^	6.94	1.24 ^ab^
1.50	9.15 ^a^	8.59 ^ab^	7.04	1.22 ^b^
2.00	9.15 ^a^	8.48 ^b^	6.84	1.24 ^ab^
2.50	9.15 ^a^	8.58 ^ab^	6.82	1.26 ^a^
SEM	0.017	0.047	0.027	0.008
*p*-value	0.001	<0.001	0.106	<0.001
Orthogonal contrast				
Control vs. L-YEP	<0.001	<0.001	0.856	<0.001
Control vs. H-YEP	<0.001	<0.001	0.554	<0.001
L-YEP vs. H-YEP	0.383	0.020	0.152	0.952
Orthogonal polynomial				
Linear	0.101	<0.001	0.514	0.001
Quadratic	0.001	<0.001	0.070	0.050
Cubic	0.001	<0.001	0.280	<0.001

SEM = standard error of the mean. YEP = a microencapsulation of Yanang leaf extract fermented with *Pediococcus acidilactici* V202, followed by entrapment within the porous structure of wheat bran. L-YEP = low level of dietary supplementation (0.50 and 1.00% YEP groups). H-YEP = high level of dietary supplementation (1.50%, 2.00%, and 2.50% YEP groups). ^a,b,c^ Different superscripts in the same column indicate significant difference (*p* < 0.05).

## Data Availability

The original contributions presented in this study are included in the article. Further inquiries can be directed to the corresponding authors.
